# Evolutionary Implications of Anoxygenic Phototrophy in the Bacterial Phylum *Candidatus* Eremiobacterota (WPS-2)

**DOI:** 10.3389/fmicb.2019.01658

**Published:** 2019-07-23

**Authors:** Lewis M. Ward, Tanai Cardona, Hannah Holland-Moritz

**Affiliations:** ^1^Department of Earth and Planetary Sciences, Harvard University, Cambridge, MA, United States; ^2^Department of Life Sciences, Imperial College London, London, United Kingdom; ^3^Cooperative Institute for Research in Environmental Sciences, University of Colorado Boulder, Boulder, CO, United States; ^4^Department of Ecology and Evolutionary Biology, University of Colorado Boulder, Boulder, CO, United States

**Keywords:** phototrophy, anoxygenic, WPS-2, Chloroflexi, reaction center

## Abstract

Genome-resolved environmental metagenomic sequencing has uncovered substantial previously unrecognized microbial diversity relevant for understanding the ecology and evolution of the biosphere, providing a more nuanced view of the distribution and ecological significance of traits including phototrophy across diverse niches. Recently, the capacity for bacteriochlorophyll-based anoxygenic photosynthesis has been proposed in the uncultured bacterial WPS-2 phylum (recently proposed as *Candidatus* Eremiobacterota) that are in close association with boreal moss. Here, we use phylogenomic analysis to investigate the diversity and evolution of phototrophic WPS-2. We demonstrate that phototrophic WPS-2 show significant genetic and metabolic divergence from other phototrophic and non-phototrophic lineages. The genomes of these organisms encode a new family of anoxygenic Type II photochemical reaction centers and other phototrophy-related proteins that are both phylogenetically and structurally distinct from those found in previously described phototrophs. We propose the name *Candidatus* Baltobacterales for the order-level aerobic WPS-2 clade which contains phototrophic lineages, from the Greek for “bog” or “swamp,” in reference to the typical habitat of phototrophic members of this clade.

## Introduction

**FIGURE 1:**
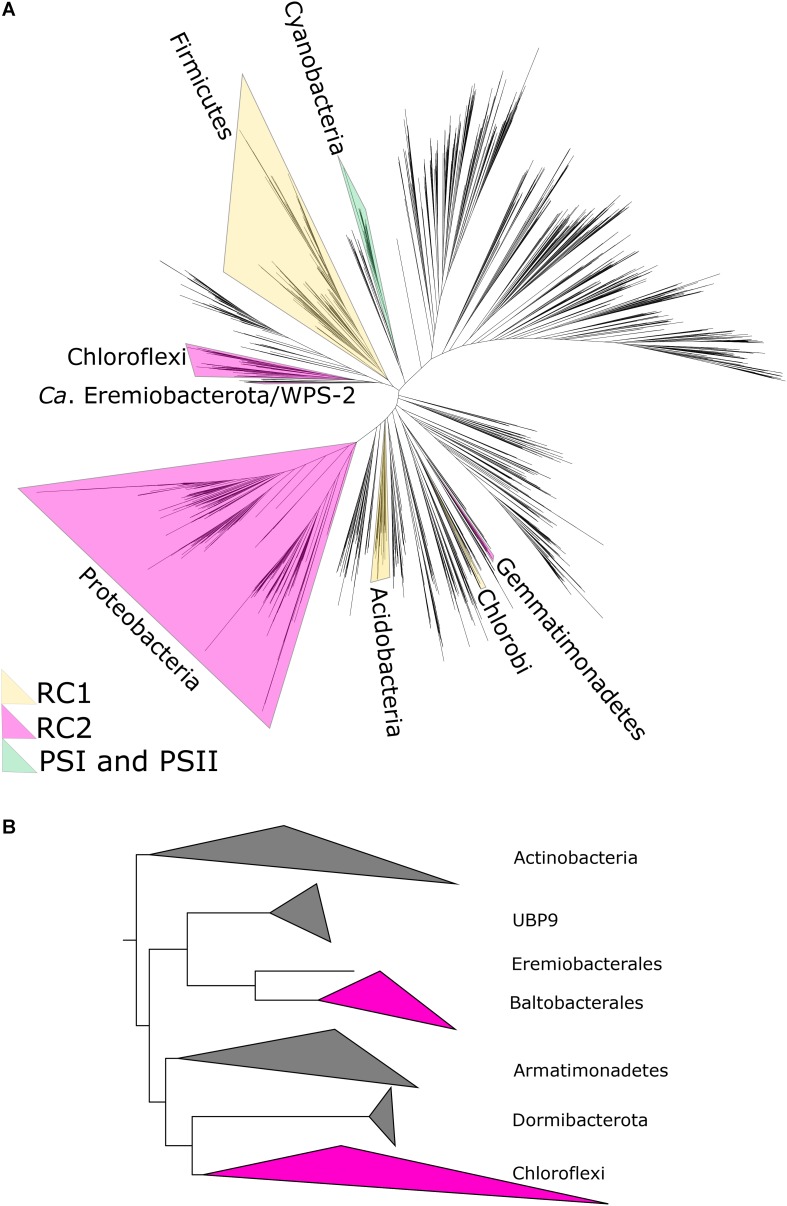
**(A)** Phylogenetic tree of bacteria showing position of WPS-2/*Ca.* Eremiobacterota, based on concatenated ribosomal protein sequences following Hug et al. (2016), with the distribution of photosynthetic reaction centers color-coded. In case where only some members of a phylum are capable of phototrophy (e.g., Heliobacteria in the much larger Firmicutes phylum), the entire phylum is highlighted for clarity. **(B)** Zoomed-in relationships of *Ca.* Eremiobacterota (here subdivided into the candidate orders UBP9, Eremiobacterales, and Baltobacterales), Chloroflexi, and other closely related phyla.

The vast majority of primary productivity on Earth is fueled by photosynthesis, both today (Raven, 2009) and through most of the history of life (Kharecha et al., 2005; Canfield et al., 2006; Ward et al., 2019b), making the organisms and proteins responsible for driving photosynthesis into critical bases for the carbon cycle. While the quantitatively most significant form of photosynthesis today is oxygenic photosynthesis, which uses water as an electron donor to power carbon fixation, there is also a great diversity of bacteria capable of anoxygenic photosynthesis using compounds such as sulfide, molecular hydrogen, ferrous iron, or arsenic as electron donors (Hohmann-Marriott and Blankenship, 2011; Thiel et al., 2018). Anoxygenic photosynthesis is restricted to members of the bacterial domain, where it has a scattered phylogenetic distribution (Figure 1). While the first observations of anoxygenic photosynthesis were made in the late 1800s (Gest and Blankenship, 2005), the known diversity of bacteria capable of anoxygenic phototrophy has exploded in recent years thanks largely to genome-resolved metagenomics (Bryant et al., 2007; Ward et al., 2018a; Figure 1). As our understanding of the physiology and evolution of phototrophy is contingent on adequate sampling of the diversity of phototrophic organisms, continuing discoveries of phylogenetically, ecologically, and biochemically novel phototrophs spurs new insights into phototrophy today and in Earth’s history.

Unlike oxygenic photosynthesis which requires the work of Type I and Type II photochemical reaction centers linked in series for water oxidation, anoxygenic phototrophs use exclusively either Type I or Type II reaction centers. Type I and II reaction centers can be differentiated by the nature of their electron acceptor (Hohmann-Marriott and Blankenship, 2011). Type I reaction centers reduce ferredoxin whereas Type II reaction centers reduce quinones. There are only five phyla of bacteria known to have phototrophic representatives that use anoxygenic Type II reaction centers: Proteobacteria, Chloroflexi, and Gemmatimonadetes (Zeng et al., 2014), recent evidence for potential phototrophy in one isolate in the Bacteroidetes (Tahon and Willems, 2017), and the newly discovered WPS-2 (Holland-Moritz et al., 2018; Figure 1). The photochemical pigments of anoxygenic Type II reaction centers are bound by two homologous subunits known as L (PufL) and M (PufM). These have traditionally been subdivided into two types, the L and M found in the reaction centers of phototrophic Proteobacteria (PbRC) and the L and M found in those of the phototrophic Chloroflexi (CfRC), with each set making deep-branching monophyletic clades (Beanland, 1990; Cardona, 2015). Therefore, substantial differences exist between the PbRC and the CfRC not only at the level of photochemistry, but also at the level of sequence identity of the core subunits, pigment and subunit composition (Xin et al., 2018; Yu et al., 2018). Phototrophic Gemmatimonadetes and Bacteroidetes, on the other hand, encode Proteobacteria-like reaction centers, suggesting that these organisms acquired phototrophy via horizontal gene transfer from members of the Proteobacteria (Zeng et al., 2014; Tahon and Willems, 2017). In addition, Proteobacterial *pufLM* genes may have also been detected in strains of the genus *Alkalibacterium* of the phylum Firmicutes from perennial springs of the high arctic (Perreault et al., 2008).

Here, we show that the genomes of the newly discovered putative phototrophs of the uncultivated candidate phylum WPS-2 (also known as *Candidatus* Eremiobacterota; Ji et al., 2017) encode a third distinct lineage of anoxygenic Type II reaction centers with novel and unusual characteristics—only the third of this type after the discovery of the Chloroflexi nearly half a century ago (Pierson and Castenholz, 1971). We also show that the WPS-2 clade encoding phototrophy is distinct from basal Eremiobacterota and related phyla in terms of traits including the capacity for aerobic respiration, membrane architecture, and environmental distribution, reflecting a divergent evolutionary history and likely novel ecological roles.

## Materials and Methods

### Metagenome Analyses

Metagenome-assembled genomes (MAGs) of WPS-2 bacteria were downloaded from NCBI WGS and JGI IMG databases. Completeness and contamination of genomes was estimated based on presence and copy number of conserved single-copy proteins by CheckM (Parks et al., 2015). Sequences of ribosomal and metabolic proteins used in analyses (see below) were identified locally with the *tblastn* function of BLAST+ (Camacho et al., 2009), aligned with MUSCLE (Edgar, 2004), and manually curated in Jalview (Waterhouse et al., 2009). Positive BLAST hits were considered to be full length (e.g., >90% the shortest reference sequence from an isolate genome) with *e*-values better than 1e^–20^. Genes of interest were screened against outlier (e.g., likely contaminant) contigs as determined by CheckM (Parks et al., 2015) and RefineM (Parks et al., 2017) using tetranucleotide, GC, and coding density content. Presence of metabolic pathways of interest was predicted with MetaPOAP (Ward et al., 2018d) to check for False Positives (contamination) or False Negatives (genes present in source genome but not recovered in metagenome-assembled genomes). Phylogenetic trees were calculated using RAxML (Stamatakis, 2014) on the Cipres science gateway (Miller et al., 2010). Transfer bootstrap support values were calculated by BOOSTER (Lemoine et al., 2018), and trees were visualized with the Interactive Tree of Life viewer (Letunic and Bork, 2016). Taxonomic assignment was confirmed with GTDB-Tk (Parks et al., 2018) and by placement in a concatenated ribosomal protein phylogeny following methods from Hug et al. (2016) Histories of vertical versus horizontal inheritance of metabolic genes was inferred by comparison of organismal and metabolic protein phylogenies to determine topological congruence (Doolittle, 1986; Ward et al., 2018a).

### Reaction Center Evolution Analyses

A total of 14 L and 12 M amino acid sequences were collected from the compiled metagenome data. The sequences were added to a dataset of Type II reaction center subunits compiled before (Cardona et al., 2019), which included sequences from Cyanobacteria, Proteobacteria, and Chloroflexi. Sequence alignments were done in Clustal Omega (Sievers et al., 2011) using 10 combined guide trees and Hidden Markov Model iterations. Maximum Likelihood phylogenetic analysis was performed with the PhyML online service^1^ (Guindon et al., 2010) using the Smart Model Selection and Bayesian information criterion for the computation of parameters from the dataset (Lefort et al., 2017). Tree search operations were performed with the Nearest Neighbor Interchange approach and the approximate likelihood-ratio test method was chosen for the computation of branch support values. A phylogenetic tree was also constructed using only L and M subunits and excluding cyanobacterial homologs.

Transmembrane helix prediction on WPS-2 L and M subunits was computed using the TMHMM Server, v. 2.0^2^ (Krogh et al., 2001) and the ΔG transmembrane helix prediction tool v. 1.0^3^ (Hessa et al., 2007). Structural homology models were carried out with SWISS-MODEL server^4^ (Waterhouse et al., 2018) using the 1.9 Å crystal structure of the Type II reaction center from the gammaproteobacterium *Thermochromatium tepidum* as template (Yu et al., 2018) (PDB ID: 5y5s). The WPS-2 L and M subunits annotated as Ga0175859_11240458 and Ga0175859_11402733, respectively, were used for the structural reconstruction.

## Results and Discussion

### Ecology and Distribution of WPS-2

Clonal libraries containing 16S rRNA sequences from the candidate WPS-2 (Wittenberg Polluted Soil) phylum were first identified in a 2001 study of soil polluted with polychlorinated biphenyl (PCB) in Germany (Nogales et al., 2001). Since then, WPS-2 has been found in 16S rRNA amplicon studies from diverse environments including acidic, polluted environments (Nogales et al., 2001; Brantner et al., 2014; Trexler et al., 2014), alpine, high latitude, and Antarctic soils (Costello, 2007; Grasby et al., 2013; Ji et al., 2016, 2017), cryoconite holes in Greenland (Stibal et al., 2015), human/canine oral microbiomes (Camanocha and Dewhirst, 2014), bogs, and peatlands (Serkebaeva et al., 2013; Bragina et al., 2015). More recently, several metagenomic studies have successfully assembled MAGs from WPS-2. These studies include samples from an acidic mine, rich in arsenic (Bertin et al., 2011); MAGs from this study were later identified as WPS-2 by Camanocha and Dewhirst (2014), Arctic (Parks et al., 2017), and Antarctic desert soils (Ji et al., 2017), boreal mosses (Holland-Moritz et al., 2018), and a peatland in Sweden (Woodcroft et al., 2018).

Although these environments are varied, several trends emerge: WPS-2 has a global distribution and is most often found in cool, acidic, and aerobic environments. While not exclusively found in low-temperature environments, most samples containing WPS-2 were collected from areas that are typically cold (i.e., Antarctica, Greenland), or undergo shorter growing seasons (high-latitude and alpine environments). Similarly, WPS-2 is more often found in sites with acidic to moderately acidic pH (between 3 and 6.5). In some cases, this acidity has come from pollution, in others, such as acidic springs, peatlands, bogs, and fir-spruce forest soil, it is a natural part of the habitat. Although WPS-2 has often been associated with polluted environments, it seems more likely that this is due to the acidity accompanying these environments than to any special resistance to the varied pollutants from these studies. Oxygen-rich environments such as mosses, cryoconite holes, and the top few centimeters of soil are typical habitat for WPS-2 and several papers have speculated that members of this phylum are therefore aerobic or microaerobic (Costello, 2007; Ji et al., 2017; Holland-Moritz et al., 2018).

Often WPS-2 makes up a minor part of the bacterial community, however, in some notable cases, the phylum has either dominated the bacterial community (e.g., 23 and 25%) (Grasby et al., 2013; Stibal et al., 2015; Ji et al., 2016) or several phylotypes from WPS-2 have been among the dominant taxa (Bragina et al., 2015; Holland-Moritz et al., 2018). Although most studies identifying WPS-2 as dominant are from specialized or unique environments (i.e., acidic springs, Antarctic soils, and cryoconite holes), an easily accessible and commonplace environment in which phylotypes of WPS-2 are often abundant is boreal mosses. Phylotypes from WPS-2 are the second-most dominant taxa across six common boreal moss species and absent in only one moss of the seven species studied (Holland-Moritz et al., 2018). Interestingly, even when mosses are not the focus of a study, WPS-2 is often more common in environments that contain them (Serkebaeva et al., 2013; Trexler et al., 2014; Bragina et al., 2015; Woodcroft et al., 2018). Currently there are no isolates of WPS-2 to investigate their biochemistry, or to use in comparative genomics, yet given their phototrophic properties and evolutionary context, such an isolate would be useful. As an accessible and abundant host to phylotypes of WPS-2, boreal mosses provide a logical starting place from which to try to culture WPS-2—an essential step in upgrading this candidate phylum to taxonomically valid status as well as for characterizing its physiology.

### Phylogenetic Context of Phototrophy in the WPS-2 Phylum

The WPS-2 phylum is located on a branch of the bacterial tree of life located near the phyla Chloroflexi, Armatimonadetes, and Dormibacteria (Figure 1). This placement has interesting implications for the evolutionary history of a variety of traits (as discussed below) but is particularly relevant for considerations of the evolutionary history of anoxygenic phototrophy.

**FIGURE 2:**
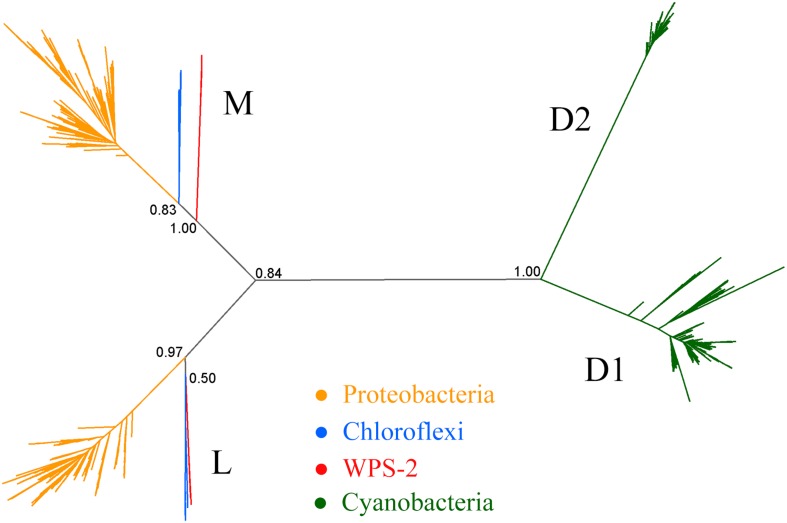
Phylogeny of Type II reaction center proteins. The tree includes L and M sequences from Proteobacteria, Chloroflexi and WPS-2, in addition to cyanobacterial D1 and D2 proteins as root.

Phylogenetic analysis of proteins for the Type II reaction center suggests that phototrophy in WPS-2 might be most closely related to that of the Chloroflexi (Figure 2, but see below). Given the relatedness of WPS-2 and Chloroflexi phototrophy proteins (Figure 2 and Supplementary Figure S1) and their relative closeness in organismal phylogenies (Figure 1), an important outstanding question is whether the last common ancestor of these phyla was phototrophic, implying extensive loss of phototrophy in both phyla and their relatives, or whether WPS-2 and Chloroflexi have independently acquired phototrophy via HGT subsequent to the divergence of these lineages (likely from an earlier branching uncharacterized or extinct group of phototrophic bacteria). While this cannot be conclusively ascertained with available data, it is apparent that aside from bacteriochlorophyll synthesis and phototrophic reaction centers, necessary proteins involved in phototrophic electron transfer (e.g., a *bc* complex or alternative complex III) are not shared, suggesting that members of these phyla independently acquired respiratory electron transfer necessary for phototrophy after their divergence. This is consistent with a relatively recent origin of phototrophy in the Chloroflexi (i.e., within the last ∼1 billion years) after the acquisition of aerobic respiration by the Chloroflexi and other phyla following the Great Oxidation Event (GOE) ∼2.3 billion years ago (Fischer et al., 2016; Shih et al., 2017b). Together with the relatively high protein sequence similarity between reaction center proteins among the phototrophic WPS-2 (discussed below) this may suggest that phototrophy in the WPS-2 has radiated on a similar or even shorter timescale.

**FIGURE 3:**
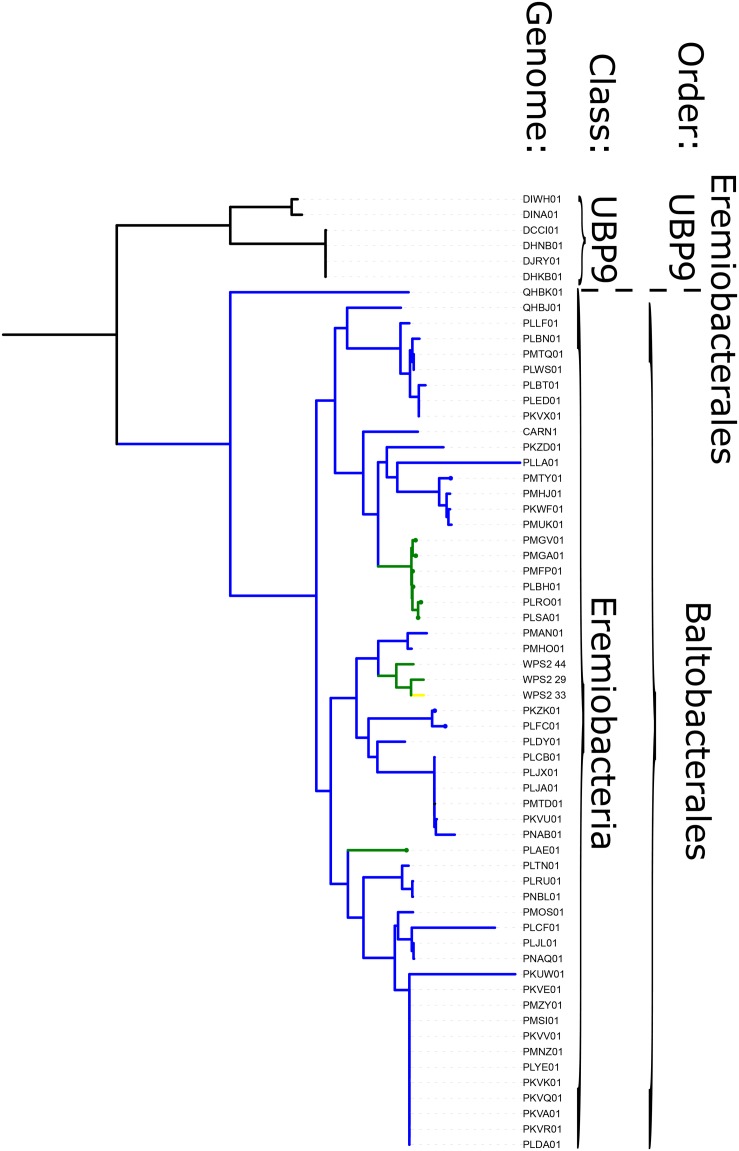
Phylogeny of WPS-2 built with concatenated ribosomal protein sequences, annotated with distribution of traits including phototrophy and aerobic respiration. Green branches encode both phototrophy and aerobic respiration; blue lineages encode aerobic respiration but not phototrophy; yellow lineages encode phototrophy but not aerobic respiration; black lineages encode neither.

Phototrophy in WPS-2 is not restricted to a single lineage, but appears in at least four discrete lineages interspersed with non-phototrophic lineages within the phylum (Figure 3). The topology of the organismal phylogeny of the WPS-2 is incongruent with that of phototrophy protein phylogenies (Figure 3 and Supplementary Figure S1), and so this distribution may best be explained by a history of HGT between members of the WPS-2 and not by a deeper ancestry of phototrophy followed by extensive loss. Similar cases of multiple HGT events of complete photosynthesis gene clusters have been demonstrated within the family Rhodobacteraceae of Alphaproteobacteria (Brinkmann et al., 2018), between classes of the phylum Chloroflexi (Ward et al., 2018a), and into the Gemmatimonadetes phylum from members of the Proteobacteria (Zeng et al., 2014), which might suggest convergent evolutionary patterns driving the distribution of Type II reaction center-based anoxygenic phototrophy within distant lineages.

Phototrophic WPS-2 appear to encode the synthesis of bacteriochlorophyll *a* (e.g., BchX, BchY, BchZ, BchF, BchC, and BchG) but no other bacteriochlorophylls (e.g., they do not encode proteins such as BchK, BchU, or BchQ for the synthesis of bacteriochlorophyll *c*, *d*, or *e*). These genomes do not appear to encode chlorosome-related proteins such as CsmA, CsmJ, or FmoA. The conversion of Mg-protoporphyrin monomethyl ester to 3,8-divinyl-protochlorophyllide *a* is an essential step in the production of (bacterio) chlorophyll compounds in all phototrophs, and can be encoded by either an aerobic (AcsF) or anaerobic (BchE) protein (Chew and Bryant, 2007). Phototrophic WPS-2 genomes all encode AcsF proteins related to those from phototrophic Chloroflexi and Acidobacteria (with the exception of WPS2_33, which is <80% complete) (Supplementary Table S1), consistent with an aerobic lifestyle. Half of the phototrophic WPS-2 genomes also encode BchE, suggesting that these strains may be facultative aerobes which are also capable of growing phototrophically in anaerobic environments.

In genomes in which phototrophy genes were recovered on contigs sufficiently long to assess clustering, bacteriochlorophyll synthesis and reaction center genes appear somewhat clustered but are spread across a larger segment of the genome and with more non-phototrophy genes intermixed than occurs for instance in phototrophic Proteobacteria and Gemmatimonadetes (Zeng et al., 2014). The gene order is fairly consistent across genomes. Supplementary Figure S7 shows three examples of the arrangements of bacteriochlorophyll synthesis genes and reaction center genes in genomes PLFC, PLAE, and PMFP. As in Chlorobi, BchC is located between the BchF and BchX in genomes PLAE and PLFC (Chew and Bryant, 2007). The reaction center proteins are within 2000 bp upstream of BchF gene in all three genomes. BchH and a BchH-like homolog are present in PLFC and PMFP as is found in phototrophic Chlorobi and Chloroflexi. In both cases the BchH-like gene is upstream of BchH. However, given the fragmented nature of metagenome assemblies, it is impossible to determine for certain the exact location and orientation of these genes in the genome.

The phylogenetic position of bacteriochlorophyll synthesis proteins including BchL of WPS-2 (Supplementary Figure S2) is somewhat in agreement with that of reaction center proteins (Figure 2 and Supplementary Figure S1). However, BchL from the phylum Chlorobi is more closely related to that of the Chloroflexi than either is to the WPS-2. This is consistent with previous studies showing that HGT of protochlorophyllide and chlorophyllide reductase between stem group phototrophic Chlorobi and Chloroflexi had occurred (Bryant et al., 2012; Sousa et al., 2013), although the direction of transfer had remained ambiguous. The new topology of BchL including WPS-2 suggests exchange of bacteriochlorophyll synthesis proteins via HGT from stem group phototrophic Chloroflexi to stem group phototrophic Chlorobi. It is likely that exchange of photosynthetic components between Chloroflexi and Chlorobi had occurred more than once, in either direction, and at different time points and may have involved a swap of reaction center type as suggested before (Bryant et al., 2012) and the acquisition of chlorosomes (Hanada et al., 2002; Garcia Costas et al., 2011).

### Additional Metabolic Traits in WPS-2 Genomes

Aside from genes encoding phototrophy, the phototroph-containing WPS-2 clade is distinct from its relatives due to a variety of metabolic traits, including proteins used for aerobic respiration and carbon fixation, as well as evidence for an outer membrane.

Most WPS-2 genomes encode proteins consistent with at least a facultative aerobic lifestyle, consistent with their typical recovery from oxic environments and presence of O_2_-using proteins such as AcsF as discussed above. The potential capacity for aerobic respiration in most WPS-2 genomes is encoded via an A-family heme-copper oxidoreductase (HCO) and a *bc* complex III. This is in contrast to the Chloroflexi, which typically encode an alternative complex III instead of a *bc* complex. Protein sequences of respiratory proteins from WPS-2 genomes primarily form single closely related clades with topologies that broadly reflect organismal relationships (Figure 3 and Supplementary Figures S3, S4); these trends suggest vertical inheritance of these genes from the last common ancestor of this clade. The main exceptions to these trends are the divergent WPS-2 genomes in the UBP9 class, which appear to be ancestrally anaerobic, and a handful of WPS-2 genomes that encode additional copies of respiratory proteins, which appear to have been acquired via horizontal gene transfer more recently and have since undergone additional HGT between members of the WPS-2. While the HCO genes in WPS-2 are interpreted here as being associated with aerobic respiration, proteins encoded by these genes are occasionally associated with O_2_ detoxification in anaerobic organisms (e.g., Ward et al., 2018c); future isolation and culture-based characterization will be necessary to confidently determine the aerobic phenotype of these organisms.

Like the Chloroflexi, phototrophic WPS-2 typically encode a B-family HCO in addition to the ancestral A-family HCO used for respiration at relatively high O_2_ concentrations (Ward et al., 2018a); the functional relationship between the B-family O_2_ reductase and phototrophy is unclear, but may relate to oxygen sensitivity of phototrophy-related proteins and the greater efficacy of B-family HCOs at low oxygen concentrations (Han et al., 2011).

While phototrophic Chloroflexi typically perform carbon fixation via the 3-hydroxypropionate bi-cycle (Shih et al., 2017b), this pathway is not encoded in any available WPS-2 genomes. Several phototrophic WPS-2 genomes encode the potential capacity for carbon fixation via the Calvin cycle including a Form I rubisco and phosphoribulokinase (Supplementary Table S1). Several other phototrophic WPS-2 genomes recovered genes for either rubisco or phosphoribulokinase but not both, which could reflect an incomplete Calvin cycle or simply the failure to recover both genes in relatively incomplete genomes. Nearly all phototrophic WPS-2 genomes encode closely related rubisco proteins related to that of *Kouleothrix aurantiaca* (Ward et al., 2018a) and *Oscillochloris trichoides* (Kuznetsov et al., 2011), with some also encoding a second copy of rubisco that belongs to the Form IA clade that includes sequences from bacteria including *Nitrosospira*, *Prochlorococcus*, and *Ectothiorhodospira* (Supplementary Figure S5; Shih et al., 2016). The phylogeny of rubisco proteins from WPS-2 genomes is not congruent with organismal or phototrophy trees, suggesting that carbon fixation may have undergone an independent history of HGT, consistent with trends in the Chloroflexi (Shih et al., 2017b; Ward et al., 2018a). Further evidence for carbon fixation having an independent history from phototrophy in WPS-2 is the presence of rubisco genes in the PMHO01 and PMHP01 genomes despite these organisms not encoding phototrophy proteins; this rubisco is not closely related to those encoded by phototrophic WPS-2, and so may reflect the independent acquisition of carbon fixation in some non-phototrophic WPS-2 lineages (perhaps to support a lithoautotrophic lifestyle).

The Chloroflexi are thought to possess a modified monoderm membrane (Sutcliffe, 2010, 2011). WPS-2 genomes recovered genes for lipopolysaccharide synthesis and outer membrane proteins such as BamA, indicating that these organisms are diderm (i.e., possess an outer membrane), reinforcing interpretations of an ancestral diderm membrane architecture followed by secondary loss of the outer membrane in the phylum Chloroflexi (Ward et al., 2017b, 2018a,b,c).

### Type II Reaction Centers in WPS-2

The phylogeny of Type II reaction center proteins showed that the L and M subunits of WPS-2 are distant, and clearly distinct, to those found in Proteobacteria and Chloroflexi (Figure 2), but the topology of the tree was unstable. It showed varying positions and levels of support for the WPS-2 sequences (Supplementary Figure S6). At the sequence identity level, the L and M in WPS-2 share 30–40% sequence identity with those in Proteobacteria and Chloroflexi. In comparison, the level of sequence identity of the Type I reaction center core subunit (PscA) of phototrophic Acidobacteria has about 39% sequence identity to that of the Chlorobi, suggesting substantial divergence. However, the overall structural comparisons do reveal a number of structural parallels to those of the Chloroflexi, in particular of the chlorosome-lacking and relatively early-branching *Roseiflexus* spp. (Xin et al., 2018).

In addition to L and M subunits, the assembled genomes of WPS-2 also contain *pufC*, *puf2A*, and *puf2C* encoding the tetraheme cytochrome direct electron donor to the oxidized special pair, and the light harvesting complex alpha and beta subunits, respectively (Holland-Moritz et al., 2018). No *puhA* was observed indicating that the reaction center of WPS-2 might lack an H subunit in a manner similar to the CfRC.

Within phototrophic WPS-2, the reaction center subunits show considerable diversity, with the level of sequence identity between the two most distant L and two most distant M subunits being about 59%. In comparison, the level of sequence identity between L or M subunits between two relatively distant strains of Chloroflexi, *Roseiflexus* spp., and *Chloroflexus* spp., is about 45% sequence identity. If the obtained sequences are representative of the diversity of phototrophic WPS-2, the greater sequence identity could potentially indicate a relatively more recent origin for their common ancestor in comparison to phototrophic Chloroflexi.

**FIGURE 4:**
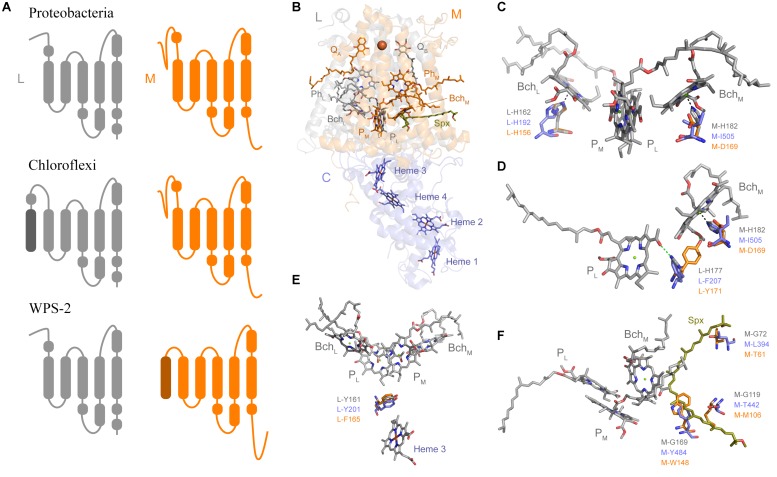
Structural comparisons of Type II reaction centers. **(A)** Schematic representation of the transmembrane helices. **(B)** The crystal structure of the PbRC from *Thermochromatium tepidum* highlighting all redox cofactors. **(C)** Comparison of the ligands to Bch_L_ and Bch_M_. Residues in gray are those from the PbRC, in blue those from the Cryo-EM structure of *Roseiflexus castenholzii*, and in orange those from the homology model of the WPS-2 reaction center proteins. **(D)** Changes around hydrogen-bonding partners to P_L_. **(E)** Changes around L-Y161, a residue important for electron transfer from the tetraheme cytochrome into the oxidized photochemical pigments. **(F)** Changes around the carotenoid-binding site. Spx denotes spirilloxanthin.

Sequence and structure prediction of the L and M subunits revealed a large number of unique characteristics (Figure 4), which is consistent with the level of distinctness seen in the phylogenetic analysis. In Proteobacteria and Cyanobacteria each Type II reaction center protein is made of 5 transmembrane helices each. The recent Cryo-EM structure of the reaction center from *Roseiflexus castenholzii* resolved a 6th helix in the L subunit (Xin et al., 2018). This was located at the N-terminus and cannot be detected through secondary structure prediction methods. In the case of WPS-2 L subunit, transmembrane helix prediction only detected the standard 5 helices; instead a novel N-terminal 6th helix was detected in the M subunit (Figure 4A).

Most of the ligands to the special pair and cofactor are conserved, but unique changes that might modulate the energetics of electron transfer can be noted. For example, in the PbRC both “monomeric” bacteriochlorophylls, Bch_L_ and Bch_M_, are coordinated by histidine ligands (Figures 4B,C). In the CfRC, a “third” bacteriopheophytin (Pierson et al., 1983; Xin et al., 2018) occupies the position of Bch_M_ and accordingly there is no histidine ligand, and instead an isoleucine is found. In WPS-2 a histidine ligand to Bch_M_ is also absent like in the CfRC, however, instead of isoleucine and strictly conserved aspartate (M-D169) is found, which may suggests the presence of bacteriochlorophyll rather than bacteriopheophytin at this position, but with modified energetics (Heimdal et al., 2007). This M-D169 might play an important role in the inactivation of electron transfer via the M branch.

In the PbRC, L-H177 provides a hydrogen bond to the special pair bacteriochlorophyll P_L_ (Figure 4D). Mutagenesis studies have shown that the strength or absence of this bond modulates the midpoint potential of the primary donor pigments P_L_ and P_M_ (Lin et al., 1994). In the CfRC, the histidine is substituted by phenylalanine, which should contribute to the lower oxidizing potential of P in comparison to the PbRC. In the CfRC the midpoint potential of the primary donor is +360–390 mV (Bruce et al., 1982; Ivancich et al., 1996; Collins et al., 2011) while in the PbRC is +450–500 mV (Moss et al., 1991; Williams et al., 1992; Woodbury and Allen, 1995; Jones, 2009). In the WPS-2 reaction center, a tyrosine is found at this position and it is potentially oriented toward Bch_M_ effectively breaking the hydrogen-bond to P_L_ like in the CfRC. The absence of the hydrogen-bond to P_L_ could lower the midpoint potential of the primary donor by about −95 mV (Lin et al., 1994), which may suggest that WPS-2 has a primary donor with a potential more similar to that of the CfRC.

In the PbRC, a carotenoid is found in contact with Bch_M_ and it is thought that this protects the system against the formation of reactive oxygen species by quenching bacteriochlorophyll triplets (Cogdell et al., 2000), see Figure 4F. In the CfRC, no carotenoid has been reported at this position and the structure from *Roseiflexus* did not show a bound carotenoid (Xin et al., 2018). In the CfRC and the WPS-2 reaction center three glycine residues, which in the PbRC give space to the carotenoid, are replaced by bulkier residues that should hinder the binding of a potential carotenoid.

In the PbRC and the CfRC a strictly conserved tyrosine (L-Y162) is located in between heme 3 (c-559) of PufC and the special pair (Figure 4E). Mutagenesis studies have shown that this tyrosine modulates the midpoint potential of both the heme and the special pair, but it is not required for fast electron transfer (Dohse et al., 1995). While a gene was identified for PufC in WPS-2 (Holland-Moritz et al., 2018), this tyrosine was found to be a phenylalanine in all WPS-2 L sequences. A deprotonation of this conserved tyrosine residue stabilizes the primary charge separation reactions (Wohri et al., 2010), therefore this mechanism is not operational in the reaction center of WPS-2.

## Perspective and Conclusion

Bacterial genomes previously assigned to WPS-2 have been assigned to a candidate phylum named *Candidatus* Eremiobacterota (Ji et al., 2017). Recent genome-resolved metagenomics has expanded the known genetic diversity of this phylum (Woodcroft et al., 2018) as well as revealing the capacity for anoxygenic phototrophy (Holland-Moritz et al., 2018). Based on the metabolic traits of various *Ca.* Eremiobacterota lineages as discussed above, together with analysis via GTDB-Tk (Parks et al., 2018; Supplementary Table S1), we propose for the aerobic WPS-2 clade that includes phototrophic members the designation of a candidate order “Baltobacterales” (from the Greek for “bog” or “swamp,” in reference to the typical habitat of these organisms) within the Eremiobacteria class of *Ca.* Eremiobacterota. Following current standards for the taxonomy of uncultured taxa (Chuvochina et al., 2019), we propose the genome WPS2_44 (IMG accession #2734482170) as the type species *Baltobacter phototrophicus* for the candidate ranks Baltobacterales (order) and Baltobacteraceae (family).

Most lineages of anoxygenic phototrophs can be preferentially found in characteristic environments unified only by the presence of light, such as anoxic regions of stratified water columns for Chlorobi (Imhoff, 2014), low-oxygen hot springs for Chloracidobacterium and phototrophic Chloroflexi (Bryant et al., 2007; Klatt et al., 2011; Hallenbeck et al., 2016; Ward et al., 2017a, 2018a), and soils for Heliobacteria (Madigan and Ormerod, 1995), though exceptions do occur e.g., Chloroflexi in carbonate tidal flats (Trembath-Reichert et al., 2016), and Heliobacteria in hot springs (Kimble et al., 1995) or soda lakes (Bryantseva et al., 1999; Asao et al., 2006). In contrast, the phototrophic Proteobacteria and Gemmatimonadetes appear to have a more cosmopolitan distribution, including freshwater, marine, and soil environments (Zeng et al., 2016; Imhoff, 2017). The preferred environment for phototrophic members of *Ca.* Eremiobacterota appears to be cold, acidic, and aerobic environments with access to sunlight and at least some members of the group forming a close association with plants, particularly mosses. This niche overlaps with that of plant-associated phototrophic Proteobacteria (Atamna-Ismaeel et al., 2012), and may be due to the relatively high oxygen tolerance of these phototrophic lineages as compared to typically more oxygen-sensitive phototrophs such as Heliobacteria and Chlorobiaceae. The apparent close and specific association of phototrophic *Ca.* Eremiobacterota with plants may reflect a long-term evolutionary association, which could further suggest that this group has radiated alongside plants over a timescale of <0.5 billion years, though this hypothesis will require molecular clocks or other analyses to test.

Most members of the class *Ca.* Eremiobacteria (i.e., the candidate orders Baltobacterales and Eremiobacterales) encode aerobic respiration using closely related A-family heme-copper oxidoreductases and *bc* complex III proteins (∼85% of all genomes, and ∼97% of >80% completeness) (Supplementary Figures S3, S4). This is in contrast to the basal UBP9 class which appears to be composed of obligate anaerobes, with no respiration genes encoded in any of the available genomes in this clade, and aerobic Chloroflexi which do not encode closely related complex III or complex IV proteins. This, together with the broad congruence of *Ca.* Eremiobacteria organismal phylogenies with complex III and complex IV protein phylogenies, implies that aerobic respiration was acquired via horizontal gene transfer into stem group *Ca.* Eremiobacteria after their divergence from UBP9 but before the radiation of crown group Eremiobacteria. It is likely that anaerobic ancestors of the Eremiobacteria and UBP9 classes diverged during Archean time, with the radiation of crown group Eremiobacteria occurring after the GOE ∼2.3 billion years ago which led to the expansion of aerobic metabolisms (Fischer et al., 2016). This hypothesis will require molecular clock estimates for the divergence and radiation of Eremiobacteria or other tests to verify, but similar evolutionary trends have been seen in other groups, including the Chloroflexi and Cyanobacteria (Fischer et al., 2016; Shih et al., 2017a, b). Following the acquisition of aerobic respiration by the last common ancestor of crown group Eremiobacteria, aerobic respiration via an A-family HCO and a *bc* complex III appears to have been largely vertically inherited, with additional respiratory proteins (e.g., second copies of *bc* complex and A-family HCOs, and the B-family HCOs associated with phototrophy) acquired via later HGT.

It is well established that all reaction centers originated as homodimers, with electron transfer occurring symmetrically on both side of the reaction center as it is still seen in Type I reaction centers (Hohmann-Marriott and Blankenship, 2008). It is thought that heterodimeric Type II reaction centers evolved from two independent duplication events, one leading to L and M in anoxygenic Type II and another leading to D1 and D2 in cyanobacterial PSII (Beanland, 1990; Sadekar et al., 2006; Cardona, 2015). The heterodimerization process led to the evolution of asymmetric electron transfer, which occurs exclusively via the L branch in anoxygenic Type II and via D1 in PSII, while the M and D2 branches became inactive, respectively. That the three different anoxygenic Type II reaction centers show distinct mechanisms of redox tuning on key positions around Bch_M_ and other photochemical pigments could indicate that these lineages started to radiate soon after the L and M duplication. Furthermore, given that anoxygenic Type II reaction centers show up to five times faster rates of evolution than cyanobacterial PSII (Cardona et al., 2019) and that the emergence of at least some of major clades of phototrophs with anoxygenic Type II reaction centers postdate the GEO (Shih et al., 2017b), it is possible that the duplication of L and M subunits, and the radiation of the known anoxygenic Type II reaction centers occurred after the origin of oxygenic photosynthesis (Cardona et al., 2019). Therefore, the idea that extant forms of anoxygenic phototrophy powered by heterodimeric Type II reaction centers represent a “primitive” form of photosynthesis is not supported by the available data. Instead, it should be considered a relatively recent, highly specialized, and mobile evolutionary innovation within the larger context of the evolution of anoxygenic photosynthesis, which reaches into Paleoarchean time (Tice and Lowe, 2004, 2006). If so, it would imply that anoxygenic photosynthesis early in Earth’s history may have been driven by now-extinct groups of bacteria using variations on phototropic pathways not yet observed today (Ward and Shih, 2019).

The known phylogenetic diversity of anoxygenic phototrophs has increased substantially in recent years, thanks largely to initial discovery of novel putative phototrophs via genome-resolved metagenomic sequencing of diverse environments (Bryant et al., 2007; Holland-Moritz et al., 2018; Ward et al., 2018a). Better coverage of the extant diversity of phototrophs and their relatives allows us to better query the evolutionary history of both organisms and their metabolisms by providing opportunities for comparative genomic, phylogenetic, and molecular clock analyses. Previous attempts to interpret the early evolution of photosynthesis relied on the narrower set of phototrophic lineages that were known at the time (Xiong et al., 2000; Raymond et al., 2002), whose analysis predated the discovery of phototrophic Chloracidobacterium, Gemmatimonadetes, Thermofonsia, and Eremiobacterota. The discovery of phototrophic Eremiobacterota and other lineages will provide crucial data for future reconsideration of the evolutionary relationships and history of phototrophy, improving our understanding of one of Earth’s most important metabolic pathways.

## Author’s Note

A version of this manuscript prior to peer review has been released as a preprint by Ward et al. (2019a).

## Data Availability

All datasets generated for this study are included in the manuscript and/or the Supplementary Files.

## Author Contributions

LW, TC, and HH-M designed the study, analyzed the data, and wrote the manuscript.

## Conflict of Interest Statement

The authors declare that the research was conducted in the absence of any commercial or financial relationships that could be construed as a potential conflict of interest.

## Footnotes

1 http://www.atgc-montpellier.fr/phyml/

2 http://www.cbs.dtu.dk/services/TMHMM/

3 http://dgpred.cbr.su.se/

4 https://swissmodel.expasy.org/
