# Supplementary material for: Evolutionary Implications of Anoxygenic Phototrophy in the Bacterial Phylum Candidatus Eremiobacterota (WPS-2)
Source: Front Microbiol. 2019 Jul 23;10:1658. doi: 10.3389/fmicb.2019.01658 (PMC6664022; doi:10.3389/fmicb.2019.01658)
Supplement: Supplementary file 1 [file Data_Sheet_1.PDF]

Supplemental Discussion
MetaPOAP False Negative estimates for the probability of phototrophy in each WPS-2 genome is found in Supplementary Table S1; genomes in which some phototrophy proteins were recovered have a high probability of encoding the complete phototrophy pathway, whereas the probability that phototrophy is encoded in other genomes but was not recovered is very low, suggesting that the distribution of phototrophy shown in Figure 3 reflects the actual distribution of phototrophy in this phylum. Only the PKZK01 genome has a low (<0.01) MetaPOAP False Negative probability of encoding a full phototrophy pathway due to encoding only a subset of necessary proteins for phototrophy despite relatively high completeness; this organism may be an instance of transitional loss of phototrophy, but this will require more complete genome sequencing or physiological data to verify.

While some Eremiobacteria genomes did not recover O_2 reductases (PMTD01, PMUE01, PNBL01, and WPS2_33), these were primarily lower completeness (<80%), were closely related to aerobic strains, and did recover a bc complex, suggesting that these genomes either encode a respiratory electron transport chain that was not recovered in the MAG or that they are undergoing secondary loss of an ancestral respiratory metabolism.

Supplementary Figure Legends
Figure S1. Protein phylogeny of PufL and PufM subunits of the Type II reaction center of WPS-2 bacteria. Protein sequences that were incomplete due to metagenome assembly (<80% of full length) omitted.

Figure S2. Protein phylogeny of BchL proteins from phototrophic bacteria. Phyla labeled, and WPS-2 highlighted in red. Protein sequences that were incomplete due to metagenome assembly (<80% of full length) omitted.

Figure S3. Protein phylogeny of A- and B-family heme-copper oxidoreductases from WPS-2, Chloroflexi, and other related phyla. WPS-2 highlighted in red. Protein sequences that were incomplete due to metagenome assembly (<80% of full length) omitted.

Figure S4. Protein phylogeny of bc complex III from WPS-2, Chloroflexi, and other related phyla. WPS-2 highlighted in red. Protein sequences that were incomplete due to metagenome assembly (<80% of full length) omitted.

Figure S5. Protein phylogeny of the large subunit of rubisco from WPS-2, Chloroflexi, and other related phyla. Major clades outside of the WPS-2 collapsed for clarity. Protein sequences that were incomplete due to metagenome assembly (<80% of full length) omitted.

Figure S6. Protein phylogeny of reaction center proteins. (A) was calculated using only L and M subunits to test for potential artefacts introduced by D1 and D2. (B) and (C) were calculated on a subset of 176 L and M sequences. (C) was calculated with the smaller dataset but removing poorly aligned regions. Poorly aligned regions were removed with Gblocks allowing for smaller final blocks, gap positions within the final blocks, and less strict flanking positions.

Figure S7. Genomic context for reaction center proteins and bacteriochlorophyll synthesis proteins in three genomes: PLFC, PLAE, and PMFP. Black lines represent the entirety of a contig, large stretches of coding DNA that do not contain reaction center or bacteriochlorophyll synthesis genes are indicated with slanted breaks. When located in the middle of a cluster, genes that are not bacteriochlorophyll synthesis or reaction center genes are indicated in gray, with putative annotations. Unless a break is indicated, genes are immediately adjacent to each other on the contig. In general, the reaction center proteins are located just up stream (within 2000 bp) of the BchF. The BchF- BchC- BchX- BchY cluster is located downstream of the reaction center proteins and, BchZ is likely part of this cluster as it immediately follows BchY in PLFC, and is located on the edges of contigs in PLAE and PMFP. BchL-BchN-BchB are located on the same contig in PLFC and PMFP and BchL is on the edge of a contig in PLAE.

Supplemental tables (Excel file)

Table S1. Table of available WPS-2 genomes, including completeness, contamination, presence of respiration and phototrophy genes, candidate phylum assignment, metagenome source, and reference information.
