# Supplementary figures and images for: Evolutionary Implications of Anoxygenic Phototrophy in the Bacterial Phylum Candidatus Eremiobacterota (WPS-2)
Source: Front Microbiol. 2019 Jul 23;10:1658. doi: 10.3389/fmicb.2019.01658 (PMC6664022; doi:10.3389/fmicb.2019.01658)

Tree scale: 0.1

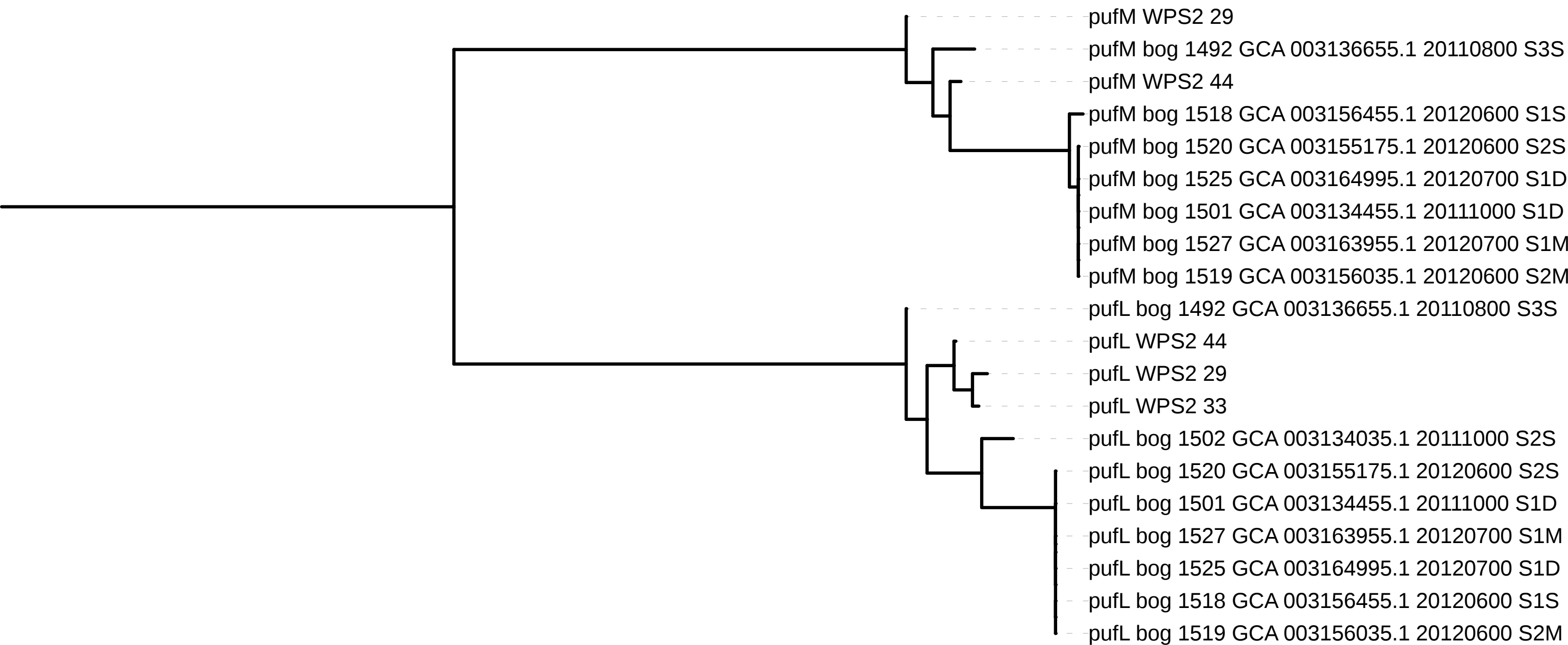

Supplement: Supplementary file 2 [file Data_Sheet_2.PDF]

Tree scale: 0.1

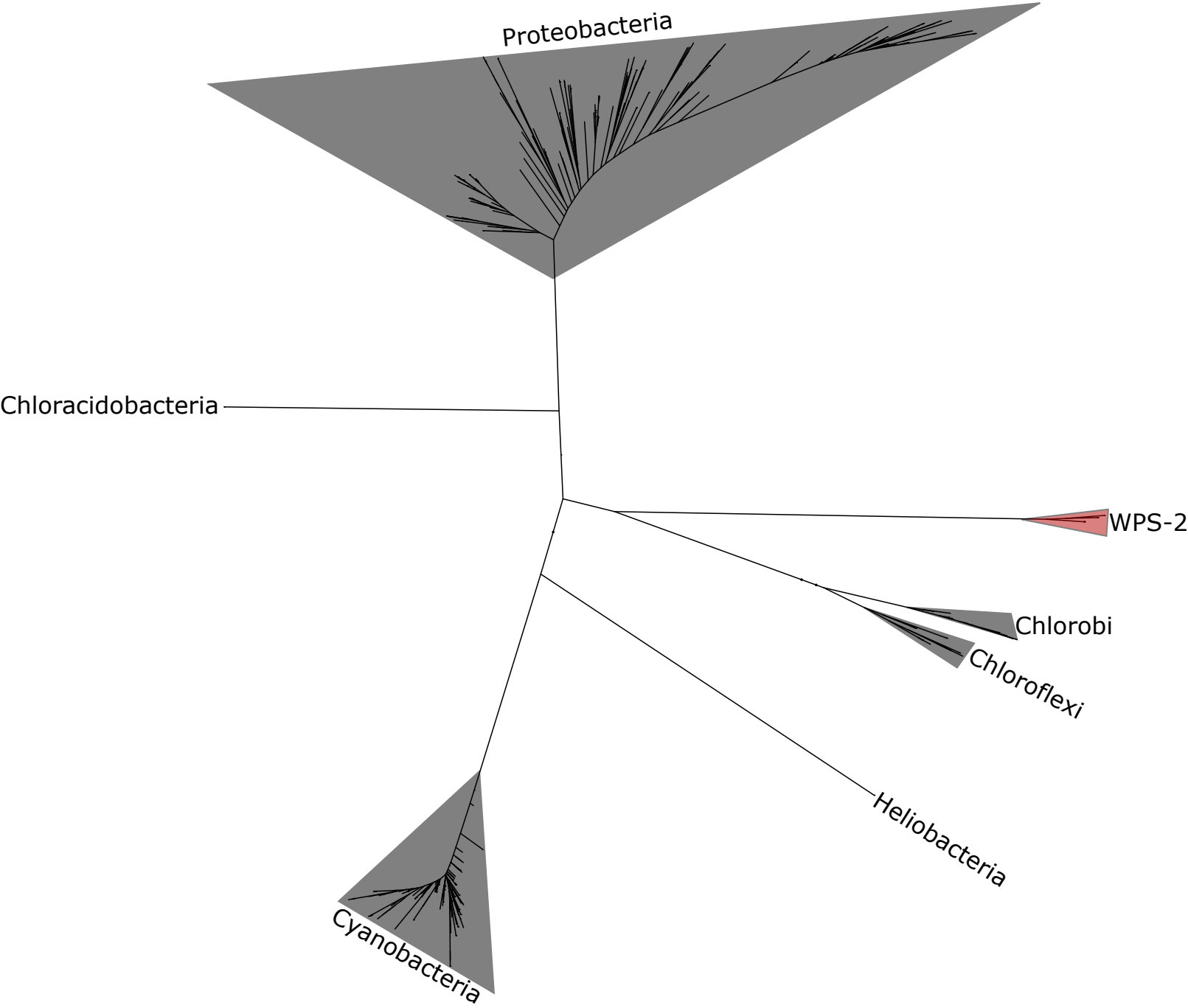

Supplement: Supplementary file 3 [file Data_Sheet_3.PDF]

Tree scale: 1

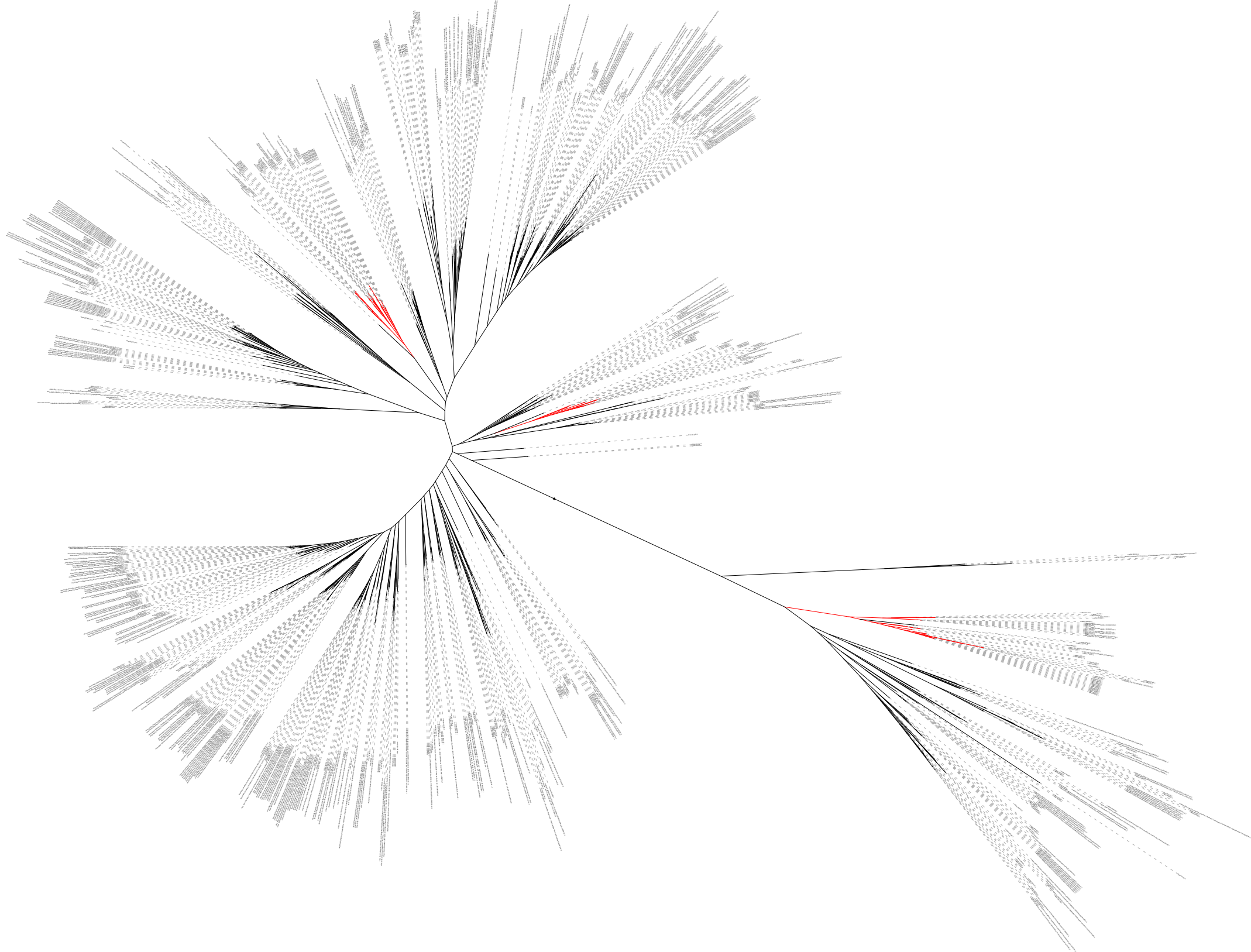

Supplement: Supplementary file 4 [file Data_Sheet_4.PDF]

Tree scale: 1

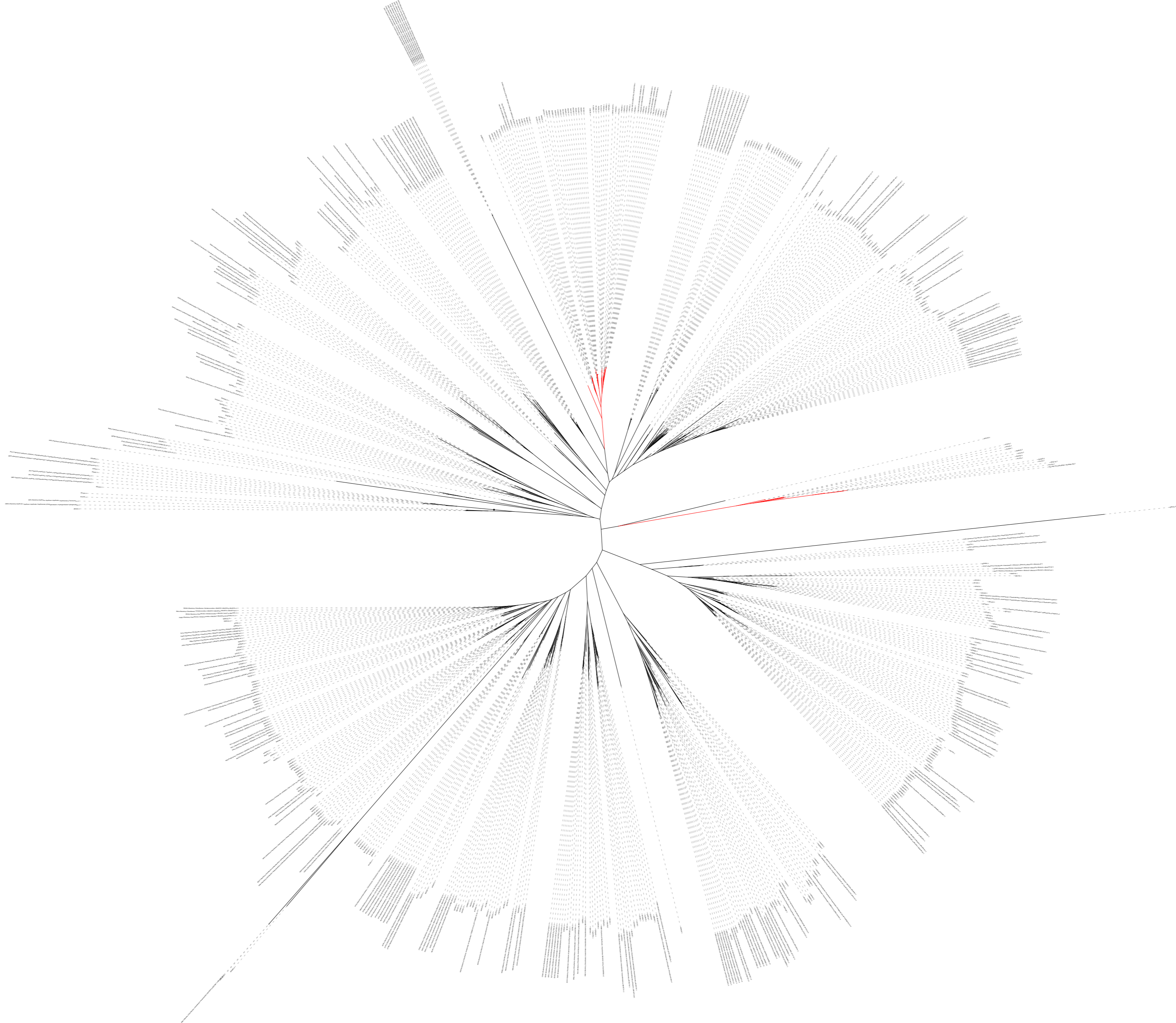

Supplement: Supplementary file 5 [file Data_Sheet_5.PDF]

A)

- Proteobacteria
- Chloroflexi
- WPS-2
- Cyanobacteria

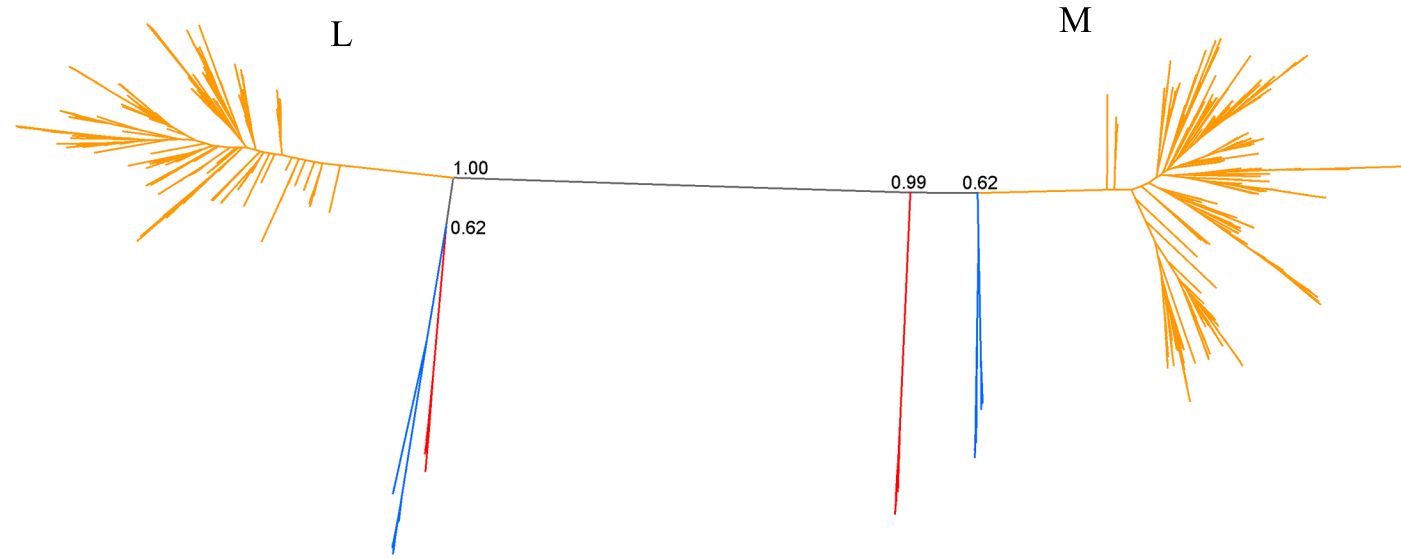

B)

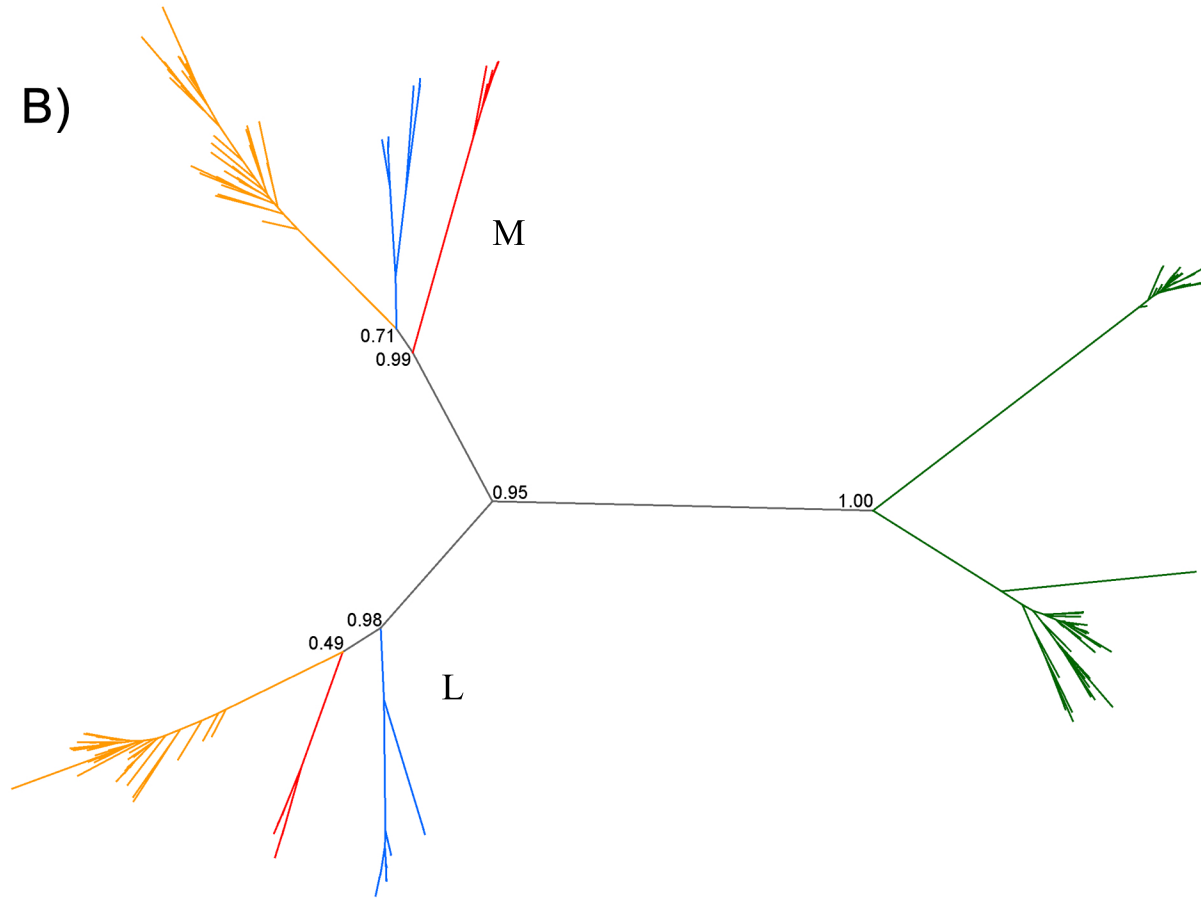

C)

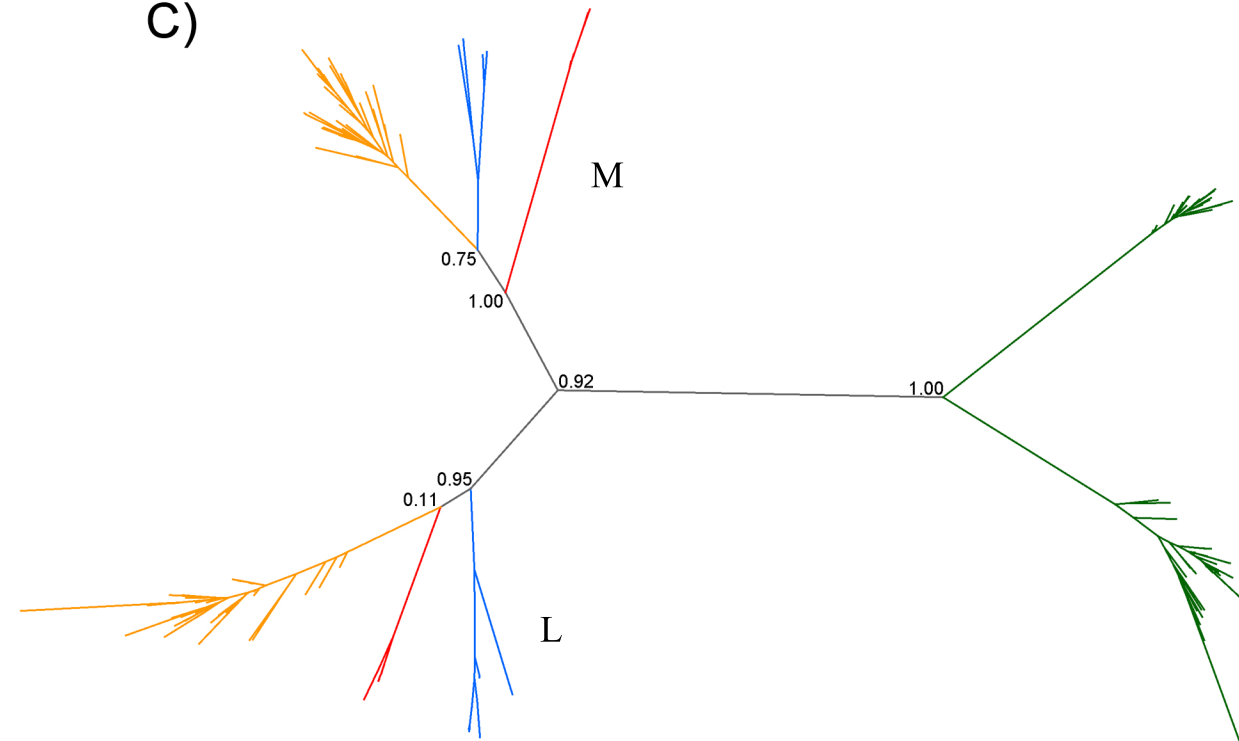

Supplement: Supplementary file 7 [file Data_Sheet_7.PDF]

Genome: PLFC (Bog\_1492)

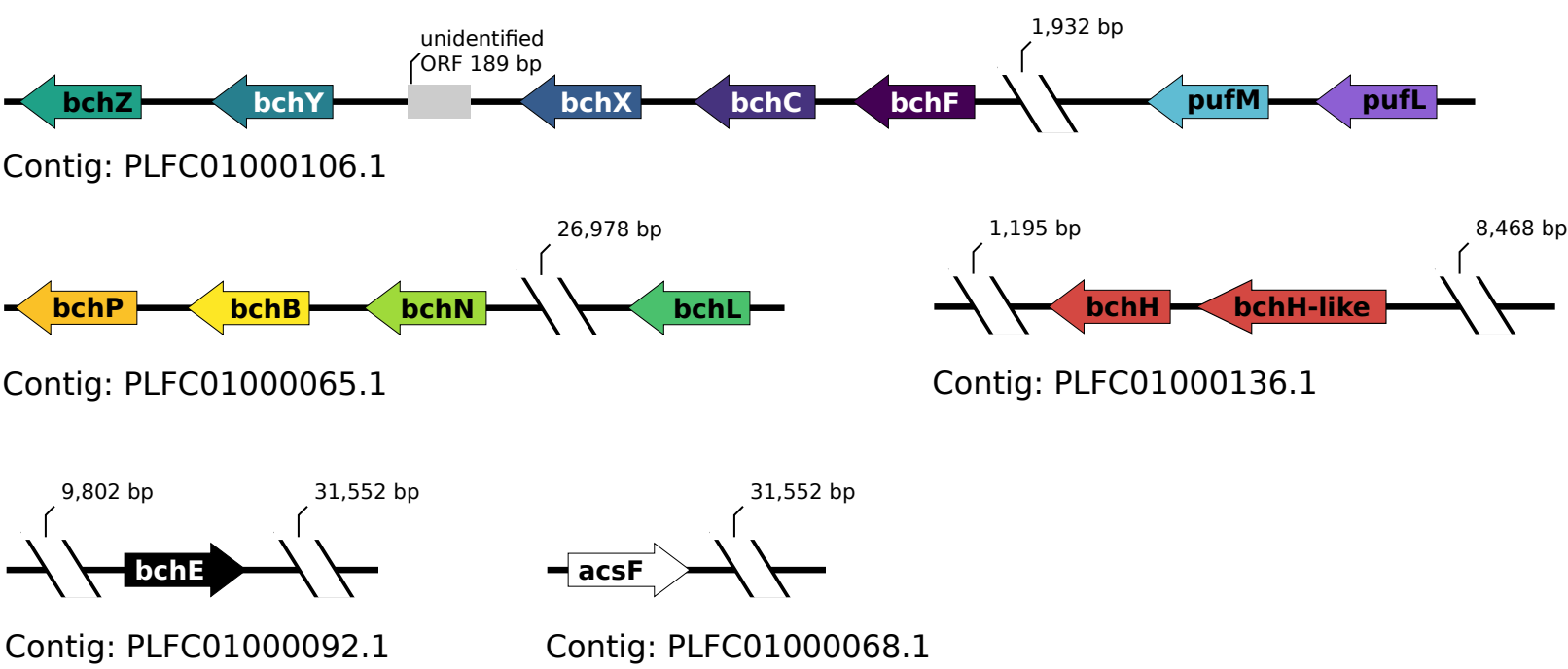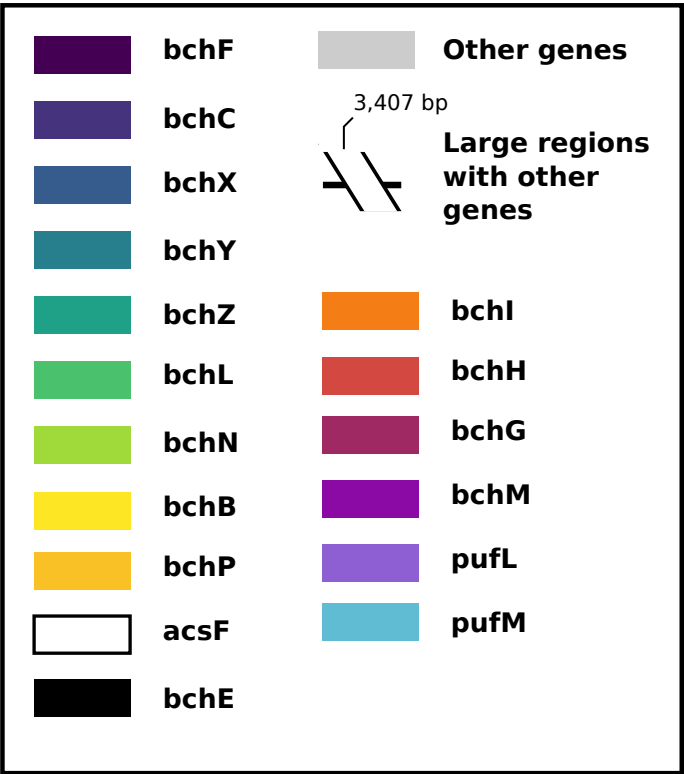

Genome: PLAE (Bog\_1502)

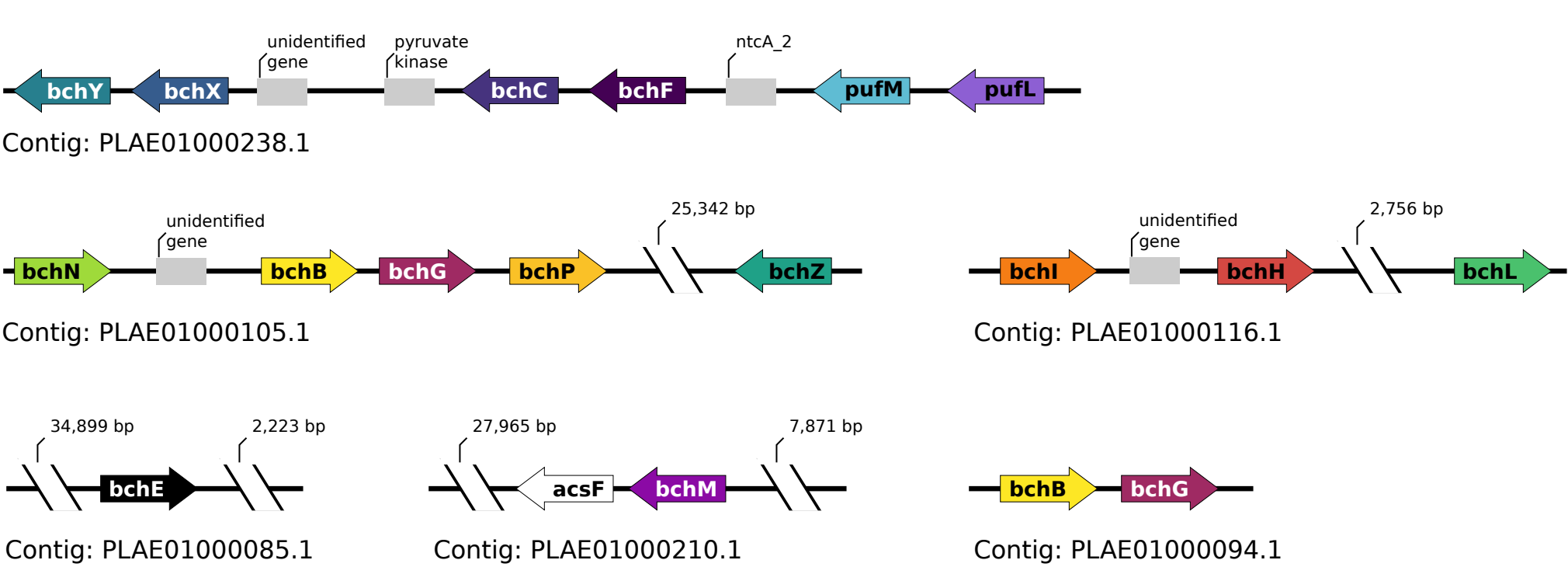

Genome: PMFP (Bog\_1520)

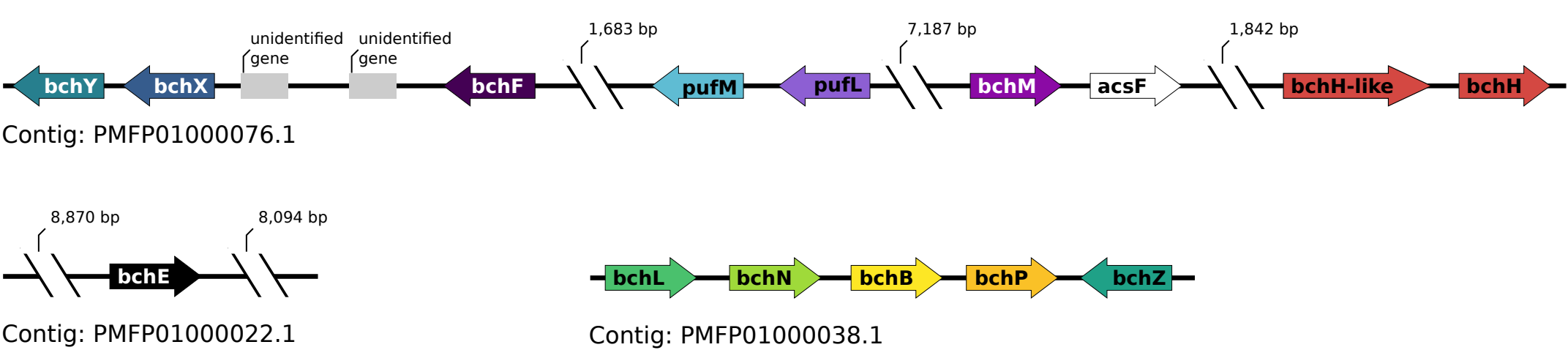

Supplement: Supplementary file 8 [file Data_Sheet_8.PDF]
