# Supplementary material for: Evolutionary Implications of Anoxygenic Phototrophy in the Bacterial Phylum Candidatus Eremiobacterota (WPS-2)
Source: Front Microbiol. 2019 Jul 23;10:1658. doi: 10.3389/fmicb.2019.01658 (PMC6664022; doi:10.3389/fmicb.2019.01658)
Supplement: Supplementary file 6 [file Data_Sheet_6.PDF]

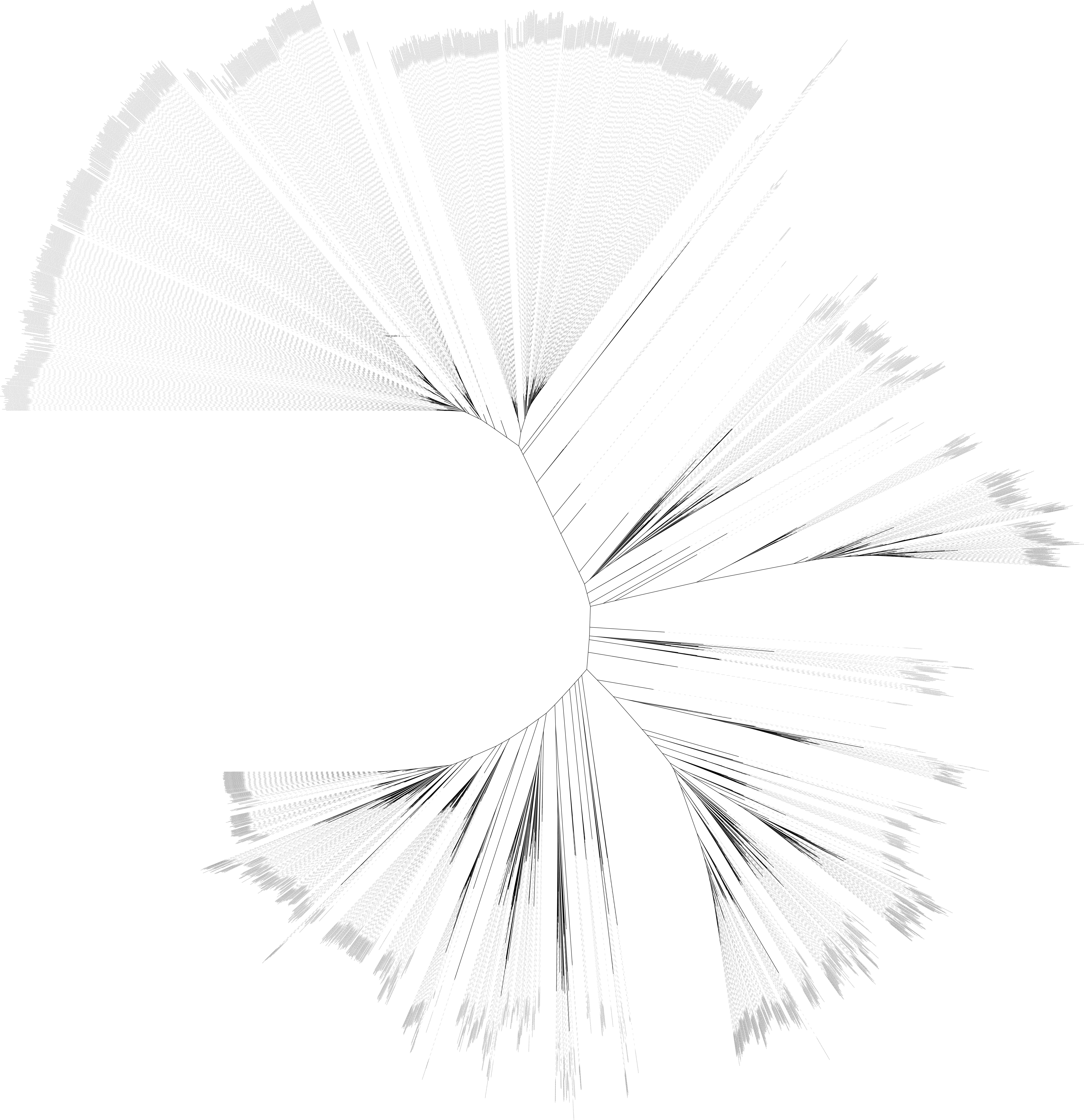
Tree scale: 1
